# Pathway Correlation Profile of Gene-Gene Co-Expression for Identifying Pathway Perturbation

**DOI:** 10.1371/journal.pone.0052127

**Published:** 2012-12-20

**Authors:** Allison N. Tegge, Charles W. Caldwell, Dong Xu

**Affiliations:** 1 Informatics Institute, University of Missouri, Columbia, Missouri, United States of America; 2 Department of Pathology and Anatomical Sciences, University of Missouri, Columbia, Missouri, United States of America; 3 Department of Computer Science and C. S. Bond Life Sciences Center, University of Missouri, Columbia, Missouri, United States of America; University of Georgia, United States of America

## Abstract

Identifying perturbed or dysregulated pathways is critical to understanding the biological processes that change within an experiment. Previous methods identified important pathways that are significantly enriched among differentially expressed genes; however, these methods cannot account for small, coordinated changes in gene expression that amass across a whole pathway. In order to overcome this limitation, we use microarray gene expression data to identify pathway perturbation based on pathway correlation profiles. By identifying the distribution of gene-gene pair correlations within a pathway, we can rank the pathways based on the level of perturbation and dysregulation. We have shown this successfully for differences between two experimental conditions in *Escherichia coli* and changes within time series data in *Saccharomyces cerevisiae*, as well as two estrogen receptor response classes of breast cancer. Overall, our method made significant predictions as to the pathway perturbations that are involved in the experimental conditions.

## Introduction

Identification of perturbed or dysregulated pathways is important for understanding changes in biological processes between two conditions. Microarray technologies are essential for identifying differences in gene expression, but there has been limited in-depth use of microarray data on a pathway level. When it comes to pathways, microarray data is typically used to identify pathways enriched with significantly differentially expressed genes. Ultimately, these studies try to extrapolate activated/repressed pathways, i.e., those pathways that show global increases and decreases in gene expression, respectively [1]. Alternatively, microarray data can also be used via co-expression networks for pathway reconstruction where little to no prior pathway knowledge is applied in the co-expression networks.

In order to identify pathways of interest, various gene set enrichment (GSE) methods are utilized [2], [3], [4], [5]. These methods rank genes by the expression’s signal-to-noise ratios [6] or the correlation of expression with the phenotype [2], determine an enrichment score for each gene ontology or pathway, and then select a set of gene ontologies or pathways based on the significance of their enrichment scores. Keller et al. extended a GSE method by utilizing dynamic programming in order to optimize this selection of significant signaling pathways [7]. GSE methods, however, require a set of genes in the gene list or pathway to be differentially expressed with statistical significance; though this requirement is sufficient in many instances, it is not necessary in order for a pathway to be dysregulated. Furthermore, this condition may not accurately reflect globally perturbed pathways. There are many biological circumstances where a few differentially expressed genes can be identified; yet large pathological differences are observed, such as diagnosis-relapse events [8], [9]. In order to help reduce this dependency on differentially expressed genes, Adewale et al. developed a regression analysis to handle pathway data, where they agglomerate a pathway-level test statistic for each individual gene in the pathway [10]. Again, though this returns a pathway level result, it still looks at each gene individually and not at how the genes coordinate with each other within the pathway. Moreover, rich information in microarray data may be underutilized. For example, current computational methods generally aggregate the biological replicates into a mean or median, thus losing added information from the available data.

Microarray data has also been used for gene-gene correlation or the co-expression of genes, which has resulted in novel pathway identification [11], [12], [13]. In particular, methods for pathway identification often rely on strong correlations between two genes. Inversely, those genes that are not co-regulated are assumed not correlated, which sometimes may not be the case. Due to these limitations, Childs et al. developed both a condition dependent and independent approach for establishing functional annotation modules to describe regulatory processes [14]. As an extension of novel pathway identification, Novak and Jain used selective gene co-expression in order to confirm valid pathways [15]. Allocco et al. also showed that there is a relationship between regulation and co-expression [16]. Through these methods, they identified that there is an increase in gene-gene correlation when a common mechanism of regulation is involved with both genes, e.g., a common transcription factor. Though these methods utilize gene-gene correlation, their goal is to identify pathways or modules that are co-regulated, yet may not interrogate general perturbations of known or unknown gene sets that are not revealed in the form of co-expression.

To aid in identifying perturbed pathways, differential gene-gene co-expression has been implemented for studying changes between different diseases and biological conditions. Lai et al. use gene-gene co-expression to identify genes with similar co-expression patterns to those that are already known to be involved in the biological process of interest [17]. This method does not rely on differential expression of genes, but relies on coordinated gene expression instead; however, it still interrogates the expression data on a gene level and does not look at the global differential gene-gene co-expression of the pathway. Cho et al. used differential co-expression to identify gene sets (e.g., pathways) that have differences in gene expression [18], but again does not look into the changing dynamics within a pathway or gene set. More applicably, however, Freudenberg et al. used differential co-expression coupled with unsupervised learning in order to identify gene sets that are significant under various conditions [19]. While their method does not utilize previously defined gene sets, it does show that given significant gene sets, there is an increase in gene-gene pair correlations. There are several disadvantages to these methods. They assess the behavior of individual genes to summarize the activity of a pathway and/or do not look at the trends of pair-wise interactions between genes within an entire predefined pathway.

To overcome these challenges, we developed a systematic method of using microarray gene expression data to identify pathway perturbation based on changes in pathway correlation profiles derived from the gene-gene pairs. Given gene sets extracted from known pathways, we identify significant pathways based on changes in gene co-expression. We can identify those pathways that are significantly perturbed in an experiment, as well as isolate groups of genes that are known to be strongly involved in a pathway’s regulation. In addition, we can identify potential significant genes that may be involved in the perturbation of a pathway but are not differentially expressed as defined by statistical confidence. Our method no longer relies on single gene involvement as well as effectively utilizes the added information gained from biological replicates within an experiment to successfully identify and rank significantly perturbed pathways.

## Results

### Pathway Ranking

Pathways were ranked based on their Benjamini corrected p-values that test if there is a significant change in the gene-gene pair correlations between two samples. Those pathways with a positive mean difference in correlations show that the gene-gene pair correlations from the treatment samples are on average higher than those gene-gene pair correlations derived from the normal samples. Conversely, those pathways with a negative mean difference show that there is a decrease in the pathway’s correlation profile under the treatment conditions. Table 1 and Table 2 show the top ranking pathways from the *E. coli* data set when comparing pH 8.7 to an ideal pH 7, and the breast cancer data set when comparing ER-positive to ER-negative, respectively (full tables in Tables S1, S2, and S3).

**Table 1:** Comparison between DAVID Gene Set Enrichment Analysis and Pathway Correlation Profile analysis of *E. coli* pH data set at pH 8.7 compared to ideal pH 7.

Pathway Correlation Profile				DAVID Gene Set Enrichment		
KEGG ID	Pathway Name	Mean Difference	p-value*	KEGG ID	Pathway Name	p-value*
ecj00780	Biotin metabolism	0.549	6.46E-20	ecj00230	Purine metabolism	1.27E-07
ecj00523	Polyketide sugar unit biosynthesis	0.688	3.09E-16	ecj00190	Oxidative phosphorylation	2.05E-07
ecj01040	Biosynthesis of unsaturated fatty acids	0.590	2.02E-14	ecj00240	Pyrimidine metabolism	1.03E-06
ecj00230	Purine metabolism	0.186	1.71E-13	ecj00340	Histidine metabolism	3.53E-05
ecj00790	Folate biosynthesis	0.445	6.28E-07	ecj00020	Citrate cycle (TCA cycle)	1.96E-04
ecj00632	Benzoate degradation via CoA ligation	−0.516	6.89E-06	ecj00620	Pyruvate metabolism	1.99E-04
ecj00053	Ascorbate and aldar	0.452	5.23E-05	ecj00500	Starch and sucrose metabolism	4.36E-04
ecj00380	Tryptophan metabolism	−0.547	7.69E-05	ecj00670	One carbon pool by folate	2.81E-03
ecj00900	Terpenoid backbone biosynthesis	0.462	4.54E-04	ecj00650	Butanoate metabolism	4.10E-03
ecj00471	D-Glutamine and D-glutamate metabolism	0.413	5.35E-04	ecj00040	Pentose and glucuronate interconversions	4.20E-03
ecj00020	Citrate cycle (TCA cycle)	−0.246	5.85E-04	ecj00010	Glycolysis/Gluconeogenesis	6.74E-03
ecj01053	Biosynthesis of siderophore group nonribosomal peptides	0.460	5.85E-04	ecj00030	Pentose phosphate pathway	7.65E-03
ecj01110	Biosynthesis of secondary metabolites	0.039	6.64E-04	ecj00052	Galactose metabolism	1.10E-02
ecj00740	Riboflavin metabolism	0.526	1.05E-03	ecj00632	Benzoate degradation via CoA ligation	2.27E-02
ecj00450	Selenoamino acid metabolism	0.433	1.12E-03	ecj00250	Alanine, aspartate and glutamate metabolism	3.03E-02

Pathway rankings based on adjusted p-values. Those pathways with positive mean differences show that the gene-gene pairs on average have a higher correlation at a stressed pH, and a lower correlation at an ideal pH. (Full pathway ranking in Supplemental Data). *: Benjamini correction.

There are 24 significantly perturbed pathways in the *E. coli* data set when looking at both pH 8.7 and pH 5 compared to the ideal pH 7 (Benjamini corrected p-value <0.01). The *S. cerevisiae* pathway mean difference and adjusted p-values were also calculated for the five time points compared to the control group (full results in Table S2). There are 16, 23, 21, 23, and 19 significantly perturbed pathways when comparing the desiccation, at 0 (dry), 15, 45, 90 and 360 minutes, to the control group, respectively (Benjamini corrected p-value <0.01). In the breast cancer data set, the pathway correlation profile method identified 33 pathways as statistically perturbed when comparing ER-positive to ER-negative patient samples (Benjamini corrected p-value <0.01).

As a comparison, a Gene Set Enrichment (GSE) analysis using DAVID was also performed [20] and the most significant KEGG pathways [21] are reported in Table 1 and Table 2 for the *E. coli* and breast cancer data sets. Of the top 15 pathways reported from DAVID for the *E. coli* data, 20% overlapped with those deemed significantly perturbed from the pathway correlation profile analysis. This resulted in the pathway correlation profile method making numerous novel predictions for perturbed pathways that were not previously discovered using GSE methods such as DAVID. For the breast cancer data set, no pathways were found in common between the two methods.

**Table 2:** Comparison between DAVID Gene Set Enrichment analysis and Pathway Correlation Profile analysis of the human breast cancer data set.

Pathway Correlation Profile				DAVID Gene Set Enrichment		
KEGG ID	Pathway Name	Mean Difference	p-value*	KEGG ID	Pathway Name	p-value*
hsa03040	Spliceosome	−0.0412	5.83E-30	hsa05219	Bladder cancer	0.004
hsa04080	Neuroactive ligand-receptor interaction	−0.0367	6.48E-22	hsa05200	Pathways in cancer	0.024
hsa03010	Ribosome	0.0382	2.74E-17	hsa04110	Cell cycle	0.069
hsa04514	Cell adhesion molecules (CAMs)	0.0298	1.63E-12	hsa04062	Chemokine signaling pathway	0.076
hsa00061	Fatty acid biosynthesis	−0.1712	3.32E-12	hsa04115	p53 signaling pathway	0.075
hsa00982	Drug metabolism - cytochrome P450	−0.058	4.79E-09	hsa00380	Tryptophan metabolism	0.063
hsa00140	Steroid hormone biosynthesis	−0.088	4.49E-08	hsa05215	Prostate cancer	0.058
hsa04060	Cytokine-cytokine receptor interaction	0.0152	5.41E-07	hsa05222	Small cell lung cancer	0.087
hsa05330	Allograft rejection	−0.0452	7.69E-07	hsa00010	Glycolysis/Gluconeogenesis	0.400
hsa00980	Metabolism of xenobiotics by cytochrome P450	−0.059	8.55E-07	hsa04144	Endocytosis	0.387
hsa03050	Proteasome	−0.0451	9.40E-07	hsa04512	ECM-receptor interaction	0.365
hsa00232	Caffeine metabolism	−0.1427	1.32E-06	hsa04114	Oocyte meiosis	0.375
hsa04740	Olfactory transduction	0.0395	1.50E-06	hsa04510	Focal adhesion	0.362
hsa05322	Systemic lupus erythematosus	0.0171	3.46E-06	hsa04960	Aldosterone-regulated sodium reabsorption	0.349
hsa04142	Lysosome	−0.0174	2.86E-05	hsa00330	Arginine and proline metabolism	0.360

Pathway rankings based on adjusted p-values. Those pathways with positive mean differences show that the gene-gene pairs on average have a higher correlation in ER-positive patient samples and a lower correlation in ER-negative patient samples for that pathway. (Full pathway ranking in Supplemental Data). *: Benjamini correction.

### Pathways Perturbed in *E. coli*

After our pathway correlation profile analysis, the *E. coli* metabolic pathways were ranked by p-values (the top 15 significant pathways shown in Table 1; full pathway results provided in Table S1). Those pathways with a mean difference greater than zero show an increase in gene-gene pair correlations under the stressed pH 8.7 when compared to an ideal pH 7.

When comparing *E. coli* at pH 8.7 against the ideal pH 7, a majority of the significant pathways (19 out of 24) show an increase in gene-gene correlations during the basic environment (p-value <0.01). Similarly, when comparing pH 5 to the ideal pH 7, only 18 pathways out of the 24 significantly perturbed pathways show this increase in gene-gene correlations under these conditions. In fact, only 14 pathways in common are significant under both stressed conditions, when compared to the ideal pH.

**Figure 1:**
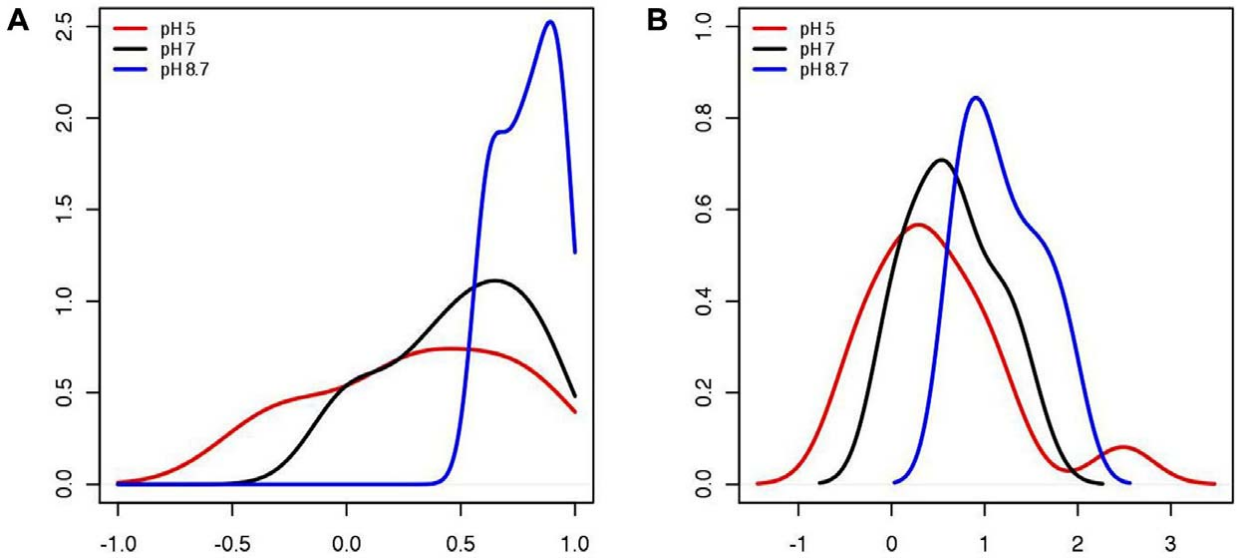
Pathway correlation profiles for Biotin Metabolism Pathway (ecj00780) in *E. coli*. (**a**) Pathway correlation profile kernel density smoothed graphs before fisher transformation of the Biotin Metabolism Pathway. (**b**) Pathway correlation profile kernel density smoothed graphs after fisher transformation of the Biotin Metabolism Pathway. (pH 8.7: blue, pH 7: black, and pH 5: red).

The Biotin Metabolism pathway (ecj00780) was the top ranked pathway based on perturbation when comparing the samples at pH 8.7 with those at pH 7. The kernel smoothed density graphs of the pathway correlation profiles at pH 5, pH 7, and pH 8.7 from the Biotin Metabolism pathway are shown in Figure 1A. The pathway correlation profile at pH 8.7 (in blue) shows an overall increase in untransformed gene-gene pair correlations within the pathway, suggesting a convergence towards a more consistent profile of the pathway during this stress. There is minimal difference between the pathway correlation profiles for this pathway at pH 5 and pH 7, also supported by the non-significant p-value. In the analysis, the Fisher transformed pathway correlation profiles for the Biotin Metabolism pathway under each condition were directly compared (Figure 1B).

### Pathways Perturbed in *S. cerevisiae*

**Figure 2:**
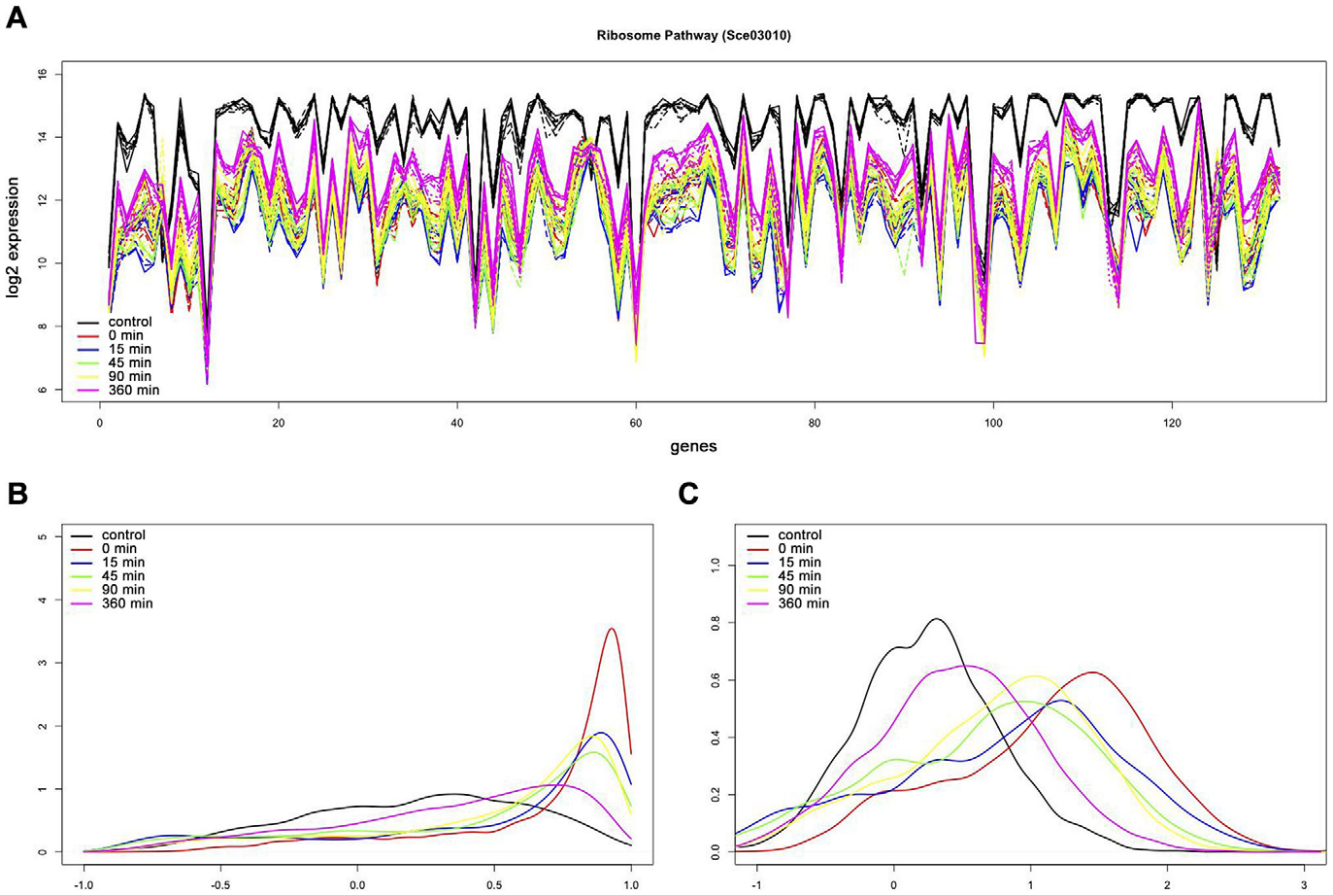
Pathway correlation profiles for Ribosome Pathway (sce03010) in *S. cerevisiae*. (**a**) Gene expression level plots of the Ribosome Pathway (**b**) Pathway correlation profile kernel density smoothed graphs before fisher transformation of the Ribosome Pathway. (**c**) Pathway correlation profile kernel density smoothed graphs after fisher transformation of the Ribosome Pathway. (Control: black, 0 minutes: red, 15 minutes: blue, 45 minutes: green, 90 minutes: yellow, and 360 minutes: magenta).

Our pathway correlation profile method compared the *S. cerevisiae* treatments (desiccated and four rehydration time points) to the control samples. Both the metabolic and non-metabolic pathways were ranked based on Benjamini corrected p-values. Complete final pathway results are in Table S2. When looking at the time series data, a few trends can be identified. Three pathways show a statistically significant increase in correlation while desiccated, and no significant changes in gene-gene correlation throughout rehydration. Six pathways show the most significant decreases in gene-gene correlation after 360 minutes of rehydration, and less significant decreases in pathway correlation at all other time points. These pathways, including the DNA replication and mRNA Surveillance pathway, show a trend towards an uncorrelated state as the rehydration process progresses (i.e. more negative mean difference). Seven pathways show significant perturbation at all time points when compared to the control sample. Of these, only the Ribosome pathway shows a convergence towards a more correlated state during all the time points. The untransformed pathway correlation profiles for the Ribosome pathway (sce03010) are shown in Figure 2B. The profile for the control sample shows a more random distribution of correlations; whereas the profiles for all the time points of rehydration and the desiccated sample show a strong skew towards a highly correlated state. Figure 2C demonstrates the pathway correlation profile distributions approaching normal after the Fisher transformation.

### Pathways Perturbed in Breast Cancer

Our pathway correlation profile analysis was performed on the ER-positive/ER-negative breast cancer data set and both the metabolic and non-metabolic pathways were ranked by adjusted p-values (top 15 significant pathways shown in Table 2; full pathway results provided in Table S3). In total, 33 out of 188 pathways were ranked as significantly perturbed (Benjamini corrected p-value <0.01) and 70% of these pathways show increases in gene-gene correlation in the ER-negative patient samples when compared to those from ER-positive patient samples.

The Spliceosome pathway (hsa03040) and the Neuroactive Ligand-Receptor Interaction pathway (hsa04080) were ranked as most perturbed when comparing the receptor status groups. Both of these pathways show an average decrease in gene-gene pair correlations when comparing ER-positive to ER-negative patient samples. Due to the larger variations among patients in cancer data sets than those in single-cell microbes (*E. coli* and *S. cerevisiae*), the gene-gene pair correlations are smaller in magnitude. Thus, the mean difference of the pathway is smaller, when compared to more controlled data sets of *E. coli* and *S. cerevisiae*.

## Discussion

Our pathway correlation profile method relies on the assumption that non-significant genes/pathways have a random correlation as their background “noise.” To show that a pathway is perturbed (i.e. activated or repressed), we need to show that the pathway no longer maintains a random gene-gene correlation profile but rather takes on a more convergent profile. This convergence could be either towards a coherently regulated state, as indicated by positive changes in correlations, or a dysregulated state, as indicated by negative changes in correlations. Final interpretations of the correlation profiles are likely to depend on the gene expression trends within the pathway.

A majority of the top 15 most significant *E. coli* pathways under basic conditions, when ranked by p-value, show an increase in correlation under the extreme conditions. This increase in correlation suggests that those pathways have a more consistently regulated system under these conditions when compared to normal; hence these pathways are likely to be universally activated or repressed in a highly coordinated manner. As for the acidic conditions, a majority of the 24 significant pathways show an increase in pathway correlation profiles, suggesting many pathways require activation/coordination of their expression under stressful conditions.

The increase in gene-gene pair correlations of the Biotin Metabolism pathway in *E. coli* at a pH of 8.7 (Figure 1), when compared to the ideal pH 7, suggests this pathway is activated at pH 8.7 and converges to a more correlated state. Biotin is a relatively unstable molecule in alkaline conditions [22], and in *E. coli* the majority of the genes in the Biotin Metabolism pathway are part of the bio-operon [23]. With a decrease in the stability, and presumably therefore the abundance of biotin under alkaline conditions, there is increased expression of the bio-operon. Since these genes are organized as an operon, increase in expression of one gene results in a coordinated increase in expression of the other genes, which can be quantified through increases in gene-gene correlations. This is confirmed by the results from the pathway correlation profile method which show an increase in gene-gene pair correlations at pH 8.7 compared to pH 7 (Figure 1A, 1B).

In the *S. cerevisiae* data set, there were varying changes in pathway correlation profiles throughout the time points within the experiment. At the time of desiccation, 16 pathways had significantly different pathway correlation profiles from those in the control (corrected p-value <0.01). Of these, eight pathways have a decrease in gene-gene correlations, and eight show an increase in gene-gene correlations, including the Ribosome pathway (sce03010) and Cell Cycle in Yeast (sce04111). Of these eight pathways, only two show this significant increase in pathway correlation profiles at the time of desiccation and no significant changes during rehydration (Cell Cycle in Yeast and Nucleotide Excision Repair). These two pathways, in essence, show increases in consistency of regulation at the time of desiccation with subsequent non-regulation during rehydration. This suggests that these pathways were necessary for cell survival at the time of desiccation but were not necessary throughout the rehydration process.

A significant increase in pathway correlation suggests:

1. the cells in the sample taken are a more homogeneous set of cells than those in the control sample; and/or
2. the pathway shows a more stable and consistent expression profile among the genes involved in this pathway, such as a regulated pathway.

These hypotheses can be shown through the Yeast Cell Cycle and the Ribosome pathways, respectively.

A more homogenous population of cells will reduce the biological variation in gene expression from positive signals, i.e., there is a stronger relationship between gene expression and phenotype [24]. According to Singh et al. the cells remained in the G_0_/G_1_ phase, or in a “holding pattern” at the time of desiccation and throughout the rehydration process [25]. The increase shown in the Cell Cycle pathway correlation at the time of desiccation suggests that there is an increase in pathway regulation stemming from a decrease in gene expression variation within this pathway. These results, together with the coincidence in cell cycle timing, suggest a more homogeneous population of cells.

Genes that are working together show increases in gene co-expression and coalesce into a more synchronous pathway [26]. The Cell Cycle pathway shows this more stable and consistent pathway correlation profile among the genes involved in this pathway. Besides showing a significant increase in correlation while desiccated, this pathway shows no significant changes at the onset of rehydration; however, it then shows a progressive move towards convergence to a more correlated state as the rehydration processes progressed, though still not significant. During this “holding pattern” time from desiccation through rehydration, the Cell Cycle pathway may not need to be orchestrated since the cells do not progress through the cell cycle. Instead, these genes show a more random background profile as would be expected from an unregulated pathway.

In contrast to the increase in pathway correlation of the Cell Cycle pathway, the DNA Replication pathway (sce03030) shows a significant decrease in gene-gene correlations at desiccation and at subsequent rehydration time points. Given that the cell population at these time points is held in the G_0_/G_1_ phase and not the S phase, there is no DNA replication occurring. All of this taken together suggests that the DNA Replication pathway is not essential for cell survival during the desiccation and rehydration, and is therefore dysregulated.

**Figure 3:**
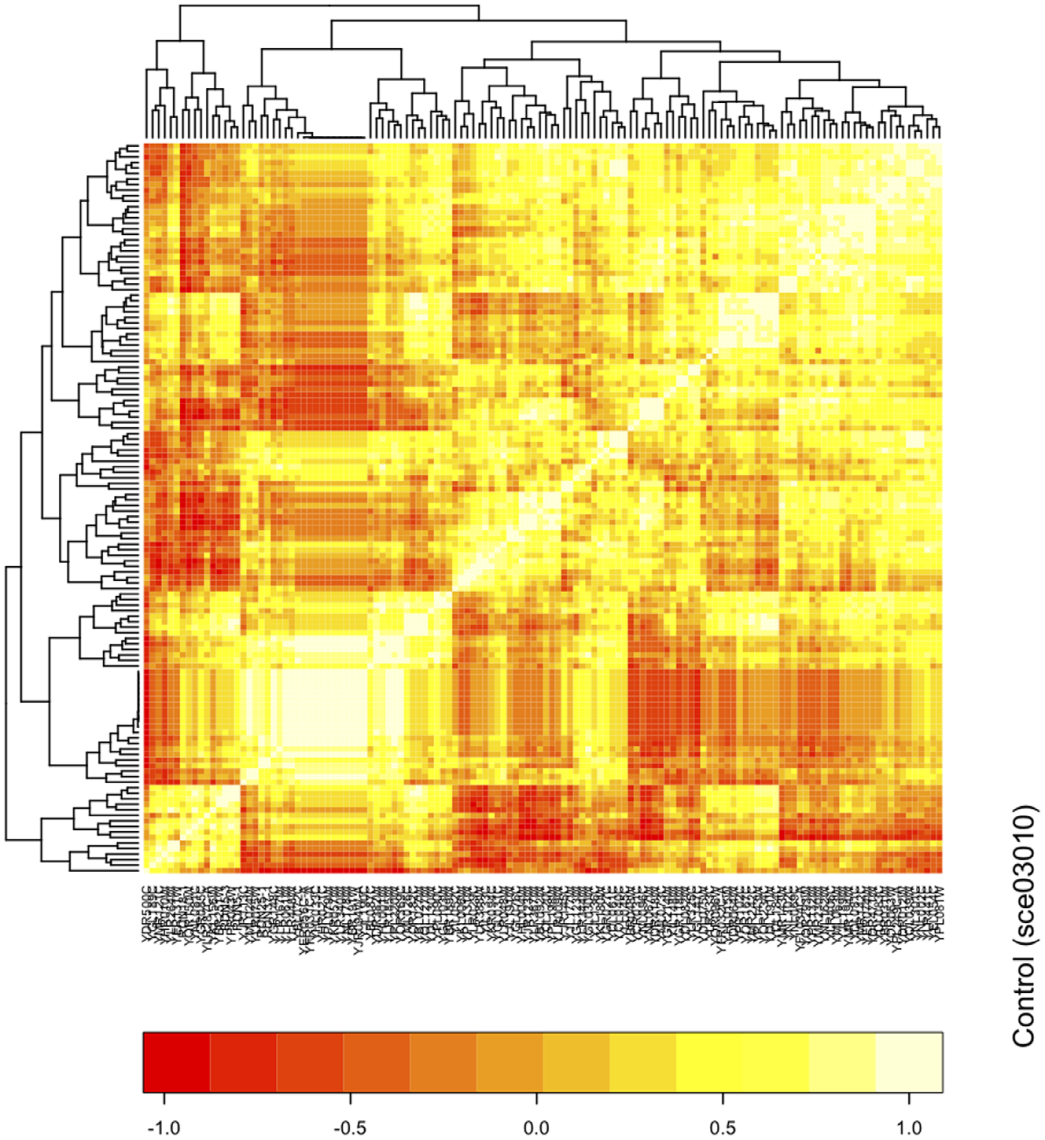
Heatmap of pathway correlation profiles for Ribosome Pathway (sce03010) in *S. cerevisiae* under control conditions. Heatmap and clustering of genes are based on their gene-gene pair correlations. Rows and columns represent genes. (Yellow: positive correlation; red: negative correlation).

The Ribosome pathway (sce03010), on the other hand, shows an increase in pathway correlation profile at all time points compared to the control. These positive correlation changes suggest that the pathway is regulated at all time points. This regulation (deactivation) can be shown through the strong, and nearly universal, decreases in gene expression within this pathway (Figure 2A). For all time points, including desiccation and throughout rehydration, the 132 genes in this pathway show a decrease in expression, averaging greater than 4-fold change when compared to the control sample’s expression profile. Given this pathway is regulated at all time points, identifying modules/clusters of genes that are coordinately regulated at each time point is important in understanding the pathway dynamics. Using a heatmap of the gene-gene pair correlations (Figure 3; Figures S1, S2, S3, S4, and S5), we can cluster the genes at each time point. Through these heatmaps, we can show that there are strong and dynamic clusters of genes that co-express together at each particular time point suggesting varying modules of regulation that are differentially activated at each time point.

Just over half of the *S. cerevisiae* pathways, however, showed no significant changes in pathway correlation profiles throughout the desiccation and rehydration process (based on corrected p-values). These pathways, including many metabolic pathways, are likely to have not changed in functional regulation or are not essential for the cell’s survival during these times. It follows that the metabolic pathways are necessary to function while the cell is still alive, and drastic changes in these pathways could result in cellular death.

In contrast, 7 out of 88 pathways (8%) show a significant change in gene-gene correlation during all time points of the experiment when compared to the control sample. All of these pathways, except the Ribosome pathway, show a decrease in gene-gene correlation throughout the desiccation and rehydration process. This decrease in gene-gene pair correlations could infer that these pathways are necessary for cell survival and/or proliferation under normal conditions, but once stresses are induced, these pathways are no longer required to be regulated during duress.

In comparing our method to the well accepted DAVID gene set enrichment method [20], there was some concordance between results on the *E. coli* pH 8.7 data. With 20% of the top 15 pathways in common, our method identifies not only significant pathways that would have been previously discovered given these experimental conditions, but also uncovers 12 additional pathways that would not previously have been investigated. The gene-gene pair correlations allow for an alternative perspective on pathway perturbation and the utilization of biological replicates independently, therefore identify significant pathways through different assumptions. The folate biosynthesis pathway (ecj00790), one of these 12 novel predictions, shows an increase in gene-gene pair correlations when comparing pH 8.7 to the control pH for *E. coli*. Within this pathway, a majority of the genes show small increases in gene expression under the basic conditions. Due to none of these increases in gene expression being statistically significant, this pathway was not reported in DAVID, a standard GSE method. It has been shown in selected species of lactic acid bacteria that higher pHs allow for increases in folate levels, suggesting more efficient folate biosynthesis under these conditions [27]. Similar explanations about folate biosynthesis could be inferred for *E. coli* under basic conditions. By exploring the data through a different perspective, i.e., our pathway correlation profiles, we can identify new pathways that have the potential to be involved in the condition and further add insight into explaining the biological mechanisms that occur within the cell when stressed at pH 8.7.

The pathway correlation profile method was used to analyze a breast cancer estrogen receptor data set. When comparing the results from our method with those from the DAVID gene set enrichment method, no pathways were found in common. In fact, only two pathways were deemed significant when using DAVID (Benjamini adjusted p-value <0.01). The discordance in predictions between our method and the DAVID method is due to the different assumptions regarding pathway perturbation as well as the DAVID method having a bias towards larger pathways, whereas our method tries to reduce pathway size biases. As a result, we can make predictions of perturbation that are independent of size.

Wang et al. reported that their gene signature for differentiating ER-positive from ER-negative patients included pathways involved in cell death, cell cycle and proliferation, DNA replication and repair, and immune response [28]. The pathway correlation profile did in fact find perturbation in the DNA Replication pathway and the Cell Cycle pathway (p-value <0.05; Table S3), and so did DAVID. The pathway correlation profile method found the Neuroactive Ligand-Receptor Interaction pathway (hsa04080) perturbed in breast cancer, but DAVID found this pathway non-significant. Within this pathway, PTGER3 is involved in many of the largest changes in gene-gene correlations. Though PTGER3 has minimal change in gene expression, the average change in Fisher transformed gene-gene correlations between this gene and all other genes in the pathway is 0.34, with increases in gene-gene pair correlations in ER-negative patient samples. Further validation is needed to show the relation between PTGER3 and estrogen receptor status in breast cancer.

Here, we have used a pathway correlation profile method to identify perturbed pathways in *E. coli, S. cerevisiae,* and a human breast cancer data set. Our method takes a global approach to analyzing gene expression data for identifying pathway perturbation. First, we take advantage of the prior knowledge of pathway members and use this to efficiently and effectively analyze the data. Second, we no longer rely on single gene involvement to identify significant pathways; rather, we look at the overall relationship between genes within a pathway and determine the level of perturbation based on changes in gene-gene relations, regardless of a specific gene’s expression profile. Third, our method exploits the biological repeats of gene expression data, while existing methods often take an average of the repeats without using the data explicitly. Lastly, our method is more robust and less influenced by the inherent noise that comes from microarrays. This method can also be adapted for additional pathway databases, such as Reactome [29], TRANSPATH [30], and pre-defined gene ontologies, as well as alternate data platforms, such as RNA-seq [31].

Our pathway correlation profile method also has some limitations. Like other computational approaches based on gene expression analysis only, our method does not include regulatory mechanisms that may not be reflected in gene expression data, such as protein translational control, post-translational modifications and kinetic control of biochemical reactions. These issues may be addressed by incorporating other types of data in the analysis. We also plan to develop a general software tool or plugin for users to apply our method easily.

## Materials and Methods

**Figure 4:**
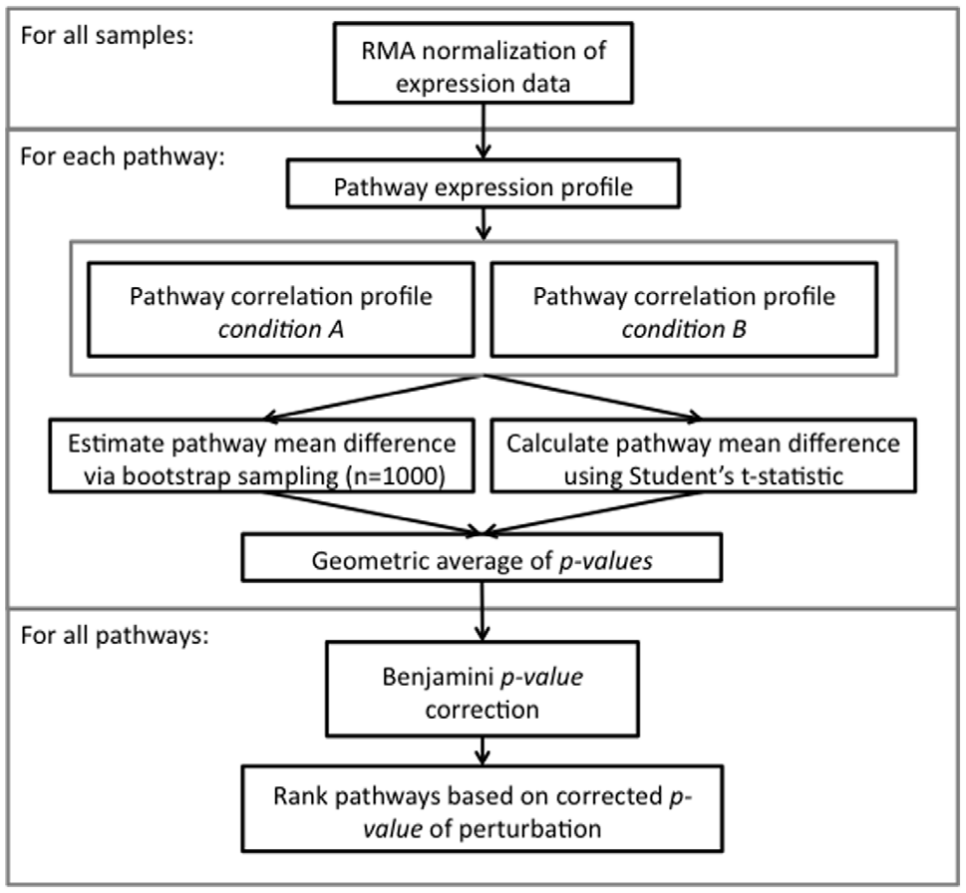
Flowchart describing pathway correlation perturbation method for analyzing gene expression data on a pathway level. Initially, gene expression data is processed and normalized. Expression profiles are then created for the set of genes involved in each pathway. Using these expression profiles, pathway correlation profiles are created in each condition for each pathway. These results are then combined to determine the pathway’s mean difference in gene-gene pair correlations, and then ranked based on their significance of perturbation.

A general schematic for our pathway correlation profile method is shown in Figure 4. Initially, gene expression data is processed and normalized. Expression profiles are then created for the set of genes involved in each pathway. Using these expression profiles, pathway correlation profiles are created for each pathway and pathway perturbation is estimated via bootstrapping. These results are then combined to rank the pathways based on their perturbation. The details for each step are further explained below.

### Expression Data

*E. coli* microarray gene expression data were downloaded from the GEO website (GSE4511) [32]. The platform for this data set is the Affymetrix *E. coli* Antisense Genome Array. This data set investigated changes in gene expression when *E. coli* was treated with different pH environments: pH 5.0, pH 7.0, and pH 8.7. In total, there were five separate samples for each pH. In addition, a *S. cerevisiae* time series data set was also downloaded from the GEO website (GSE1311-4) [33]. This data set utilized the Affymetrix Yeast S98 arrays. Singh et al. performed a desiccation in combination with rehydration of *S. cerevisiae* in order to identify transcriptional changes over time. To determine changes over the rehydration process, they performed a time series experiment with nine samples at each of the following time points: 0 (dry), 15, 45, 90 and 360 minutes after rehydration. Samples from a control group were also included. To show the robustness of this method, a breast cancer data set comparing gene expression between positive and negative estrogen receptor (ER-positive and ER-negative) status patients was analyzed [28]. Breast cancer gene expression data set was downloaded from the GEO website (GSE2034) and is from Affymetrix Human U133a GeneChips with 77 ER-negative and 209 ER-positive patient samples.

### Pathway Data

Pathway data were collected from the KEGG database [21], including the metabolic pathway files for *E. coli*, and both the metabolic and non-metabolic pathway files for *S. cerevisiae* and breast cancer data sets. Each xml file was parsed using custom scripts, and the genes involved in the pathway were identified and used as the pathway genes. Those pathways with fewer than five genes were removed from the analysis to avoid statistical insignificance. A total of 64, 88, and 188 *E. coli*, *S. cerevisiae*, and *H. sapiens* pathways met this criterion, respectively, and were used in this analysis.

### Expression Profiles

The microarray gene expression data were normalized using the Robust Multi-Array average expression measure (RMA) function from the *affy* package in R [34]. The expression profile, *E*_i_, for gene i is represented as:
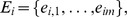
where *e*_im_ is the mean expression value of all probe sets for gene *i* on chip *m*. Gene expression profiles were created for each gene in a pathway, and each expression value was the log_2_ value for the normalized array intensity values.

In the breast cancer data set, noisy probes were removed. To accomplish this, those probes that were above the median of the chip in at least a quarter of the arrays were retained for the analysis. RMA was subsequently used to normalize the remaining probes.

### Pathway Correlation Profiles

Pathway correlation profiles (e.g., correlation matrix) were created for each pathway in the data set. The profiles are calculated for all gene pairs among different chips at a given condition using Pearson correlations and are represented as:
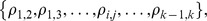


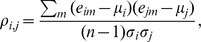
where *k* is the number of genes in the pathway, *m* is the chip index, *n* is the total number of chips in a sample, 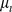 and 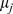 are the mean expression values for gene *i* and *j*, respectively, and 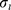 and 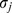 are the standard deviations, respectively. The pathway correlation profiles were calculated individually for each pH in *E. coli*, each time point in *S. cerevisiae*, and the ER-positive and ER-negative samples, respectively. Due to sample size biases in the breast cancer data set, the gene-gene pair correlations in the ER-positive class were estimated by repeatedly sampling 77 chips randomly and taking the final average of gene-gene pair correlations. Lastly, to ensure that the gene-gene pair correlations have a normal distribution and stable variance, the pathway correlation profiles were transformed using the Fisher transformation.

### Pathway Ranking

The derived pathway correlation profiles were used to rank the pathways based on most significant perturbation for each condition. In order to quantify the differences in correlation of specific gene-gene pairs between two conditions, the pathway perturbation was considered the average of these changes in correlation. Initially, a paired t-test was performed where each gene-gene pair correlation at one condition was directly compared to the corresponding correlation under the other condition. The paired t-test between condition (1) and condition (2) follows:
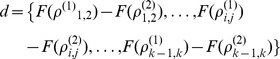
where



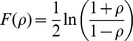
and



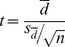
where *d* is the paired differences in Fisher transformed gene-gene pair correlations, 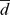 is the sample mean of the differences in transformed gene-gene pair correlations, 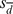 is the standard deviation of *d*, and *n* is the number of gene-gene pairs in the pathway. Due to a bias towards pathways of larger size, bootstrapping was then implemented separately in order to estimate the average change in gene-gene pair correlation for each pathway. For this, 100 gene-gene pairs were randomly sampled from each pathway and the average change in gene-gene pair correlations was calculated. From these samplings, the mean change in gene-gene pair correlations can be estimated using a z-score. We then combined the p-values from the Student’s t-test analysis and bootstrapping, correct for multiple testing by using a Benjamini correction, and rank the pathways by corrected p-values. In particular, the p-values from the bootstrapping process remove the biases due to sizes of pathways. This method differs from the standard gene expression analysis in two ways: (1) we utilize all biological replicates as opposed to assessing the mean expression of individual genes, and (2) we calculate the changes in gene-gene correlation between conditions rather than calculating a change in expression between conditions.

## Supporting Information

Figure S1 **Heatmap of pathway correlation profiles for Ribosome Pathway (sce03010) in** ***S. cerevisiae*** **at 0 minutes (dry).** Heatmap and clustering of genes are based on their gene-gene pair correlations. Rows and columns represent genes. (Yellow: positive correlation; red: negative correlation).
(TIFF)

Figure S2 **Heatmap of pathway correlation profiles for Ribosome Pathway (sce03010) in** ***S. cerevisiae*** **at 15 minutes.** Heatmap and clustering of genes are based on their gene-gene pair correlations. Rows and columns represent genes. (Yellow: positive correlation; red: negative correlation).
(TIFF)

Figure S3 **Heatmap of pathway correlation profiles for Ribosome Pathway (sce03010) in** ***S. cerevisiae*** **at 45 minutes.** Heatmap and clustering of genes are based on their gene-gene pair correlations. Rows and columns represent genes. (Yellow: positive correlation; red: negative correlation).
(TIFF)

Figure S4 **Heatmap of pathway correlation profiles for Ribosome Pathway (sce03010) in** ***S. cerevisiae*** **at 90 minutes.** Heatmap and clustering of genes are based on their gene-gene pair correlations. Rows and columns represent genes. (Yellow: positive correlation; red: negative correlation).
(TIFF)

Figure S5 **Heatmap of pathway correlation profiles for Ribosome Pathway (sce03010) in** ***S. cerevisiae*** **at 360 minutes.** Heatmap and clustering of genes are based on their gene-gene pair correlations. Rows and columns represent genes. (Yellow: positive correlation; red: negative correlation).
(TIFF)

Table S1 **Full Pathway Correlation Profile analysis of** ***E. coli*** **pH data set at all pH comparisons.**
(XLSX)

Table S2 **Full Pathway Correlation Profile analysis of** ***S. cerevisiae*** **data set at all time point comparisons with the control conditions.**
(XLS)

Table S3 **Full Pathway Correlation Profile analysis of breast cancer data set comparing ER positive with ER negative patient samples.**
(XLS)
