# Supplementary figures and images for: Pathway Correlation Profile of Gene-Gene Co-Expression for Identifying Pathway Perturbation
Source: PLoS One. 2012 Dec 20;7(12):e52127. doi: 10.1371/journal.pone.0052127 (PMC3527387; doi:10.1371/journal.pone.0052127)

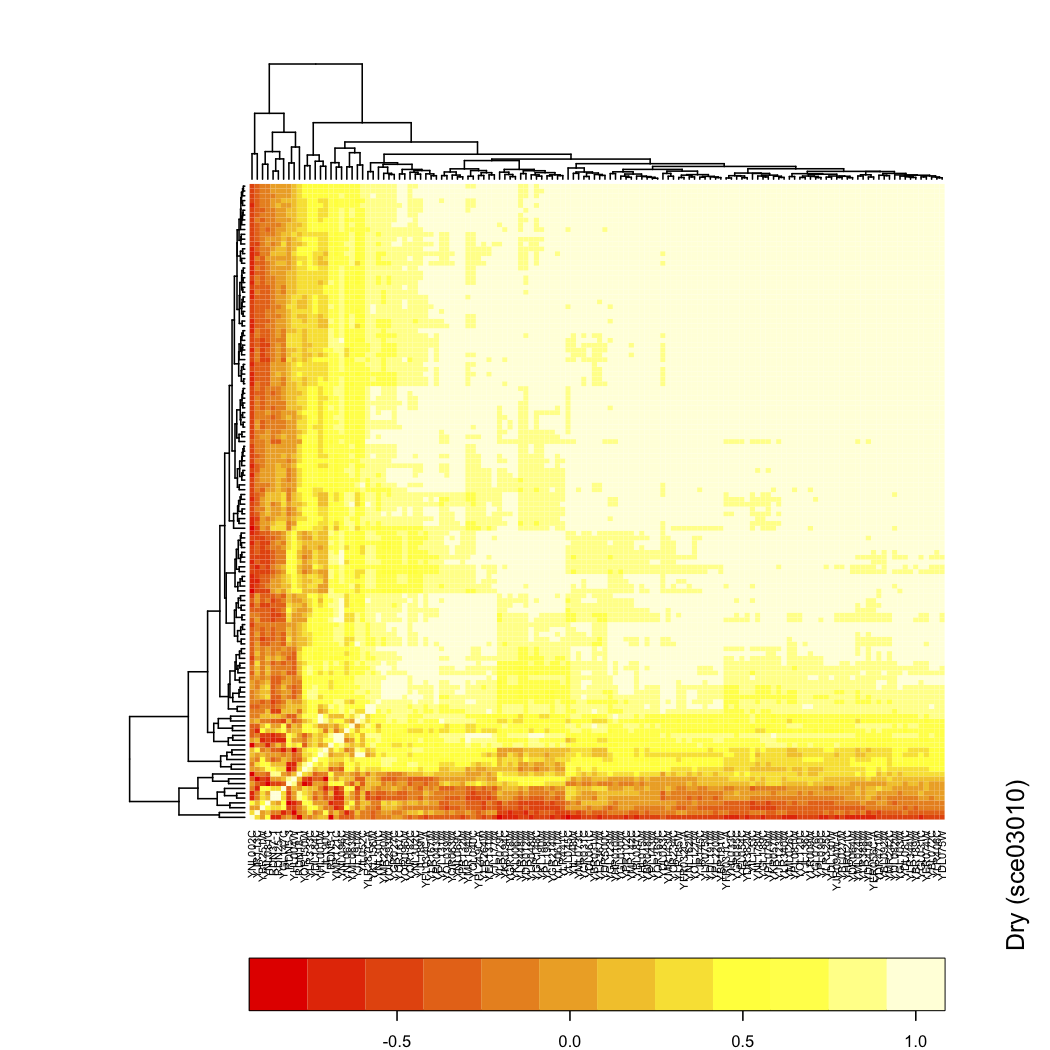

Supplement: Figure S1 — Heatmap of pathway correlation profiles for Ribosome Pathway (sce03010) in S. cerevisiae at 0 minutes (dry). Heatmap and clustering of genes are based on their gene-gene pair correlations. Rows and columns represent genes. (Yellow: positive correlation; red: negative correlation). (TIFF) [file pone.0052127.s001.tiff]

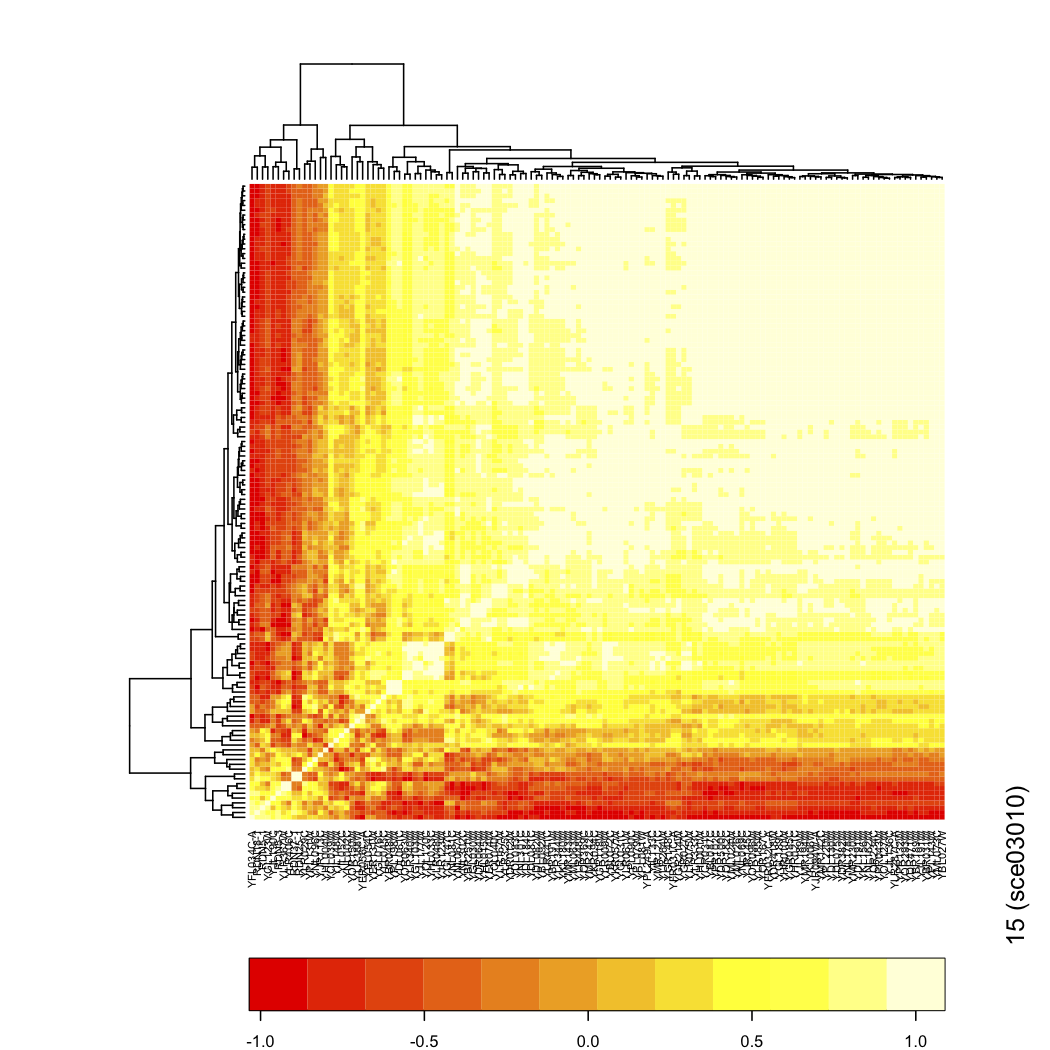

Supplement: Figure S2 — Heatmap of pathway correlation profiles for Ribosome Pathway (sce03010) in S. cerevisiae at 15 minutes. Heatmap and clustering of genes are based on their gene-gene pair correlations. Rows and columns represent genes. (Yellow: positive correlation; red: negative correlation). (TIFF) [file pone.0052127.s002.tiff]

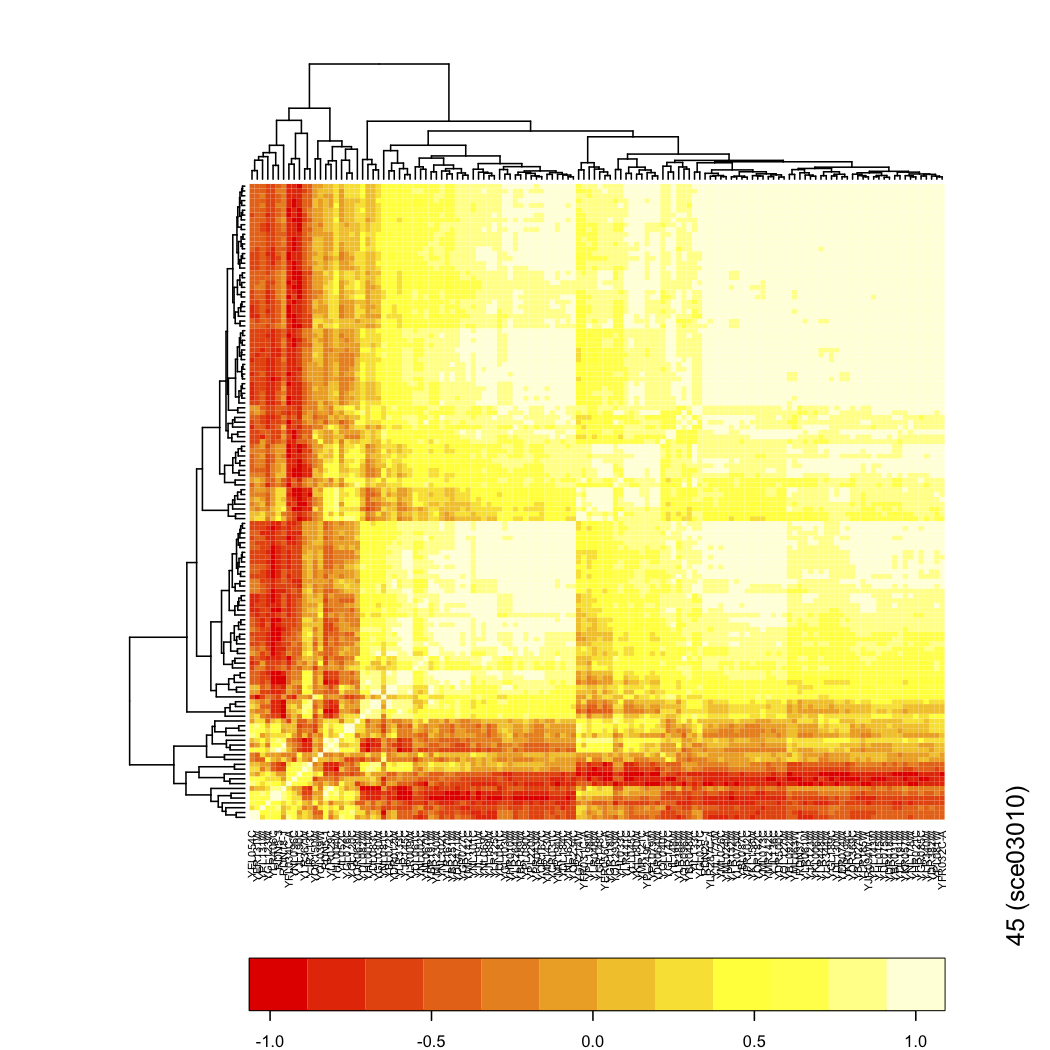

Supplement: Figure S3 — Heatmap of pathway correlation profiles for Ribosome Pathway (sce03010) in S. cerevisiae at 45 minutes. Heatmap and clustering of genes are based on their gene-gene pair correlations. Rows and columns represent genes. (Yellow: positive correlation; red: negative correlation). (TIFF) [file pone.0052127.s003.tiff]

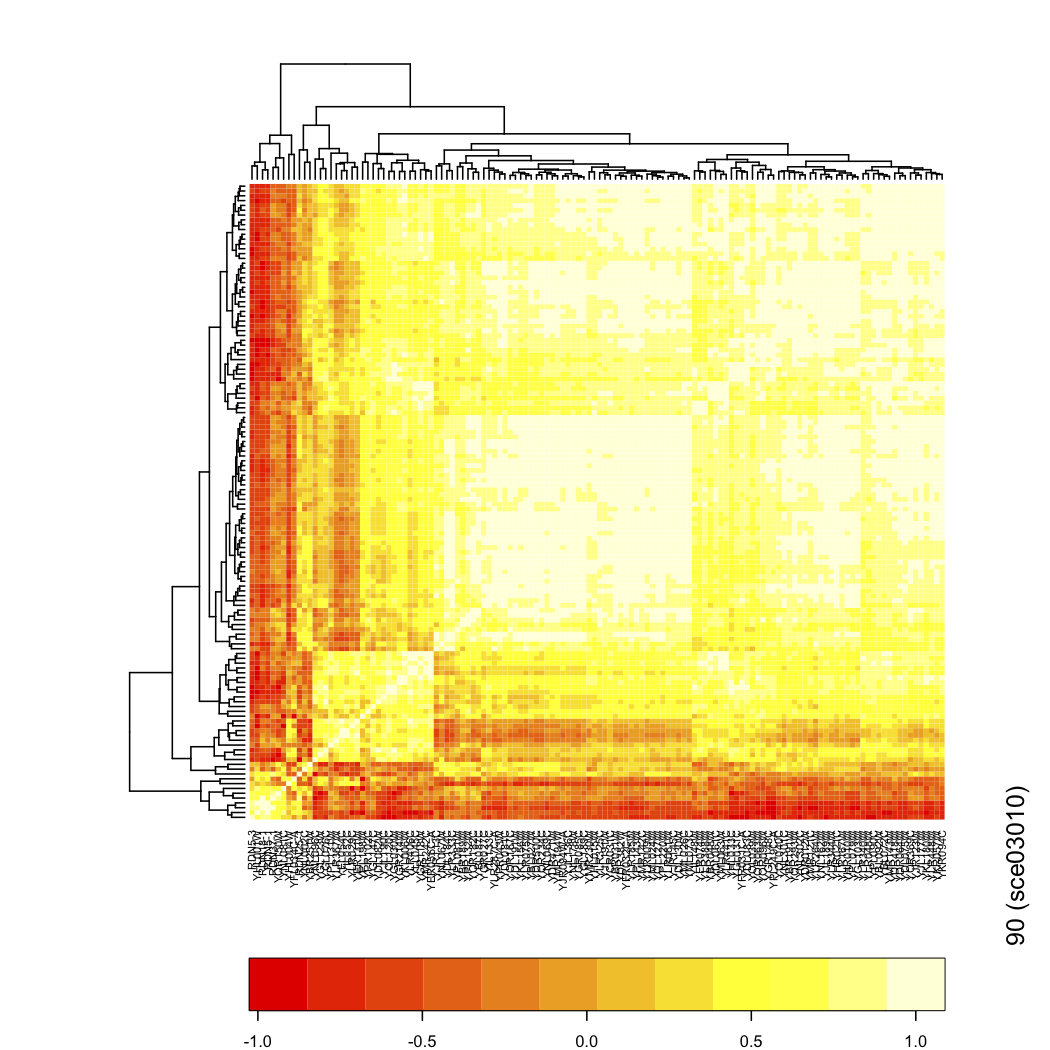

Supplement: Figure S4 — Heatmap of pathway correlation profiles for Ribosome Pathway (sce03010) in S. cerevisiae at 90 minutes. Heatmap and clustering of genes are based on their gene-gene pair correlations. Rows and columns represent genes. (Yellow: positive correlation; red: negative correlation). (TIFF) [file pone.0052127.s004.tiff]

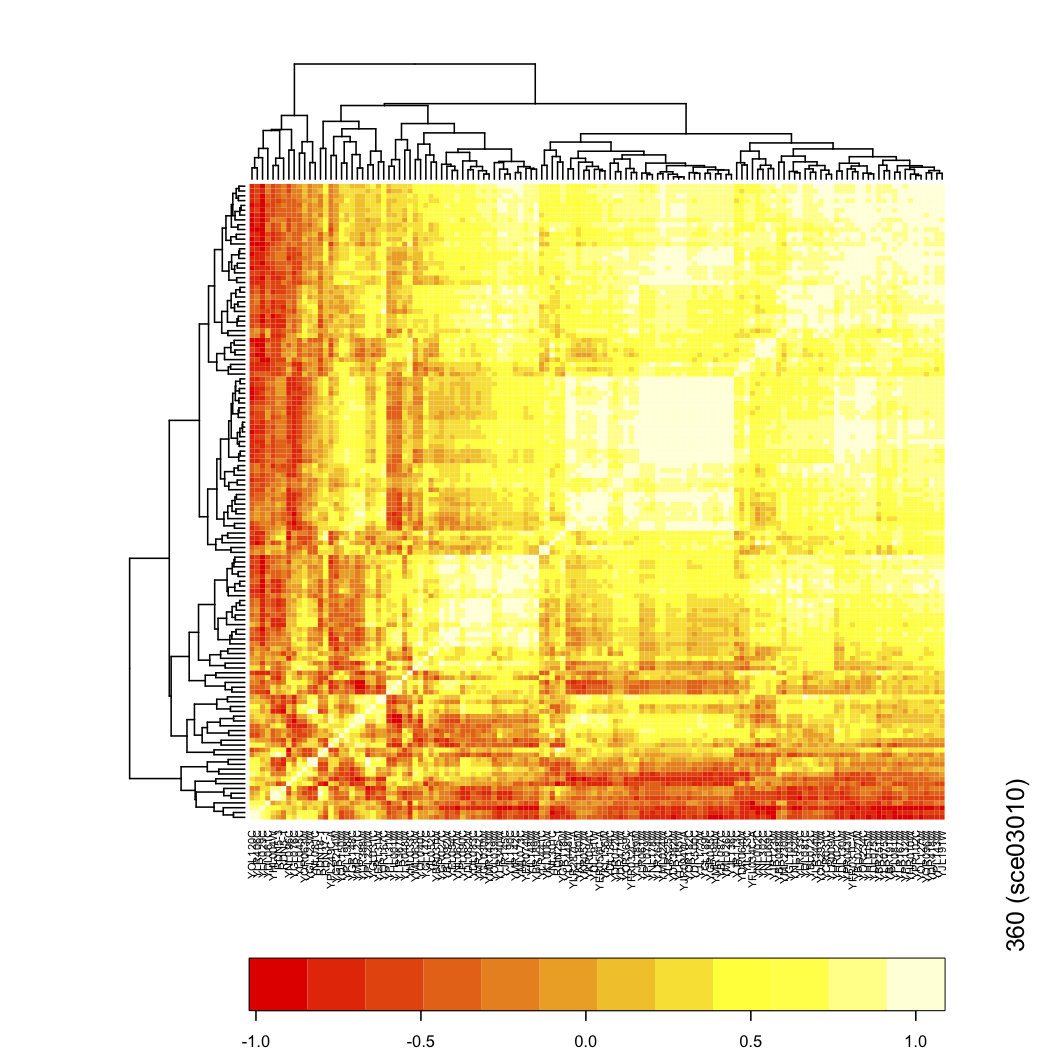

Supplement: Figure S5 — Heatmap of pathway correlation profiles for Ribosome Pathway (sce03010) in S. cerevisiae at 360 minutes. Heatmap and clustering of genes are based on their gene-gene pair correlations. Rows and columns represent genes. (Yellow: positive correlation; red: negative correlation). (TIFF) [file pone.0052127.s005.tiff]
